# Three-dimensional rotational angiography in children with an aortic coarctation

**DOI:** 10.1007/s12471-016-0899-2

**Published:** 2016-09-22

**Authors:** N. L. P. Starmans, G. J. Krings, M. M. C. Molenschot, F. van der Stelt, J. M. P. J. Breur

**Affiliations:** Department of Paediatric Cardiology, Wilhelmina Children’s Hospital, University Medical Center Utrecht, Utrecht, The Netherlands

**Keywords:** Three-dimensional imaging, Angiography, Aortic coarctation, Endovascular procedures, Paediatrics, Congenital heart defects

## Abstract

**Background:**

Children with aortic coarctations (CoA) are increasingly percutaneously treated. Good visualisation of the CoA is mandatory and can be obtained with three-dimensional rotational angiography (3DRA). This study aims to compare the diagnostic and therapeutic additional value of 3DRA with conventional biplane angiography (CA) in children with a CoA.

**Methods:**

Patients undergoing percutaneous treatment of CoA with balloon angioplasty (BA) or stent between 2003 and 2015, were retrospectively reviewed on success rate, complications, radiation and technical settings. Diagnostic quality of CA and 3DRA and additional value of 3DRA were scored.

**Results:**

In total, 134 patients underwent 183 catheterisations, 121 CA and 62 3DRA-guided. Median age was 0.52 years in the BA group and 11.19 years in the stent group. 3DRA was superior to CA in displaying the left ventricle (*p* = 0.008), ascending aorta (*p* < 0.001), aortic arch (*p* = 0.005) and coronary arteries (*p* < 0.001). In the BA group, 3DRA had a significantly higher success rate than CA (100.0 % versus 68.9 %, *p* = 0.016). All stent interventions were successful. Complication rates did not differ significantly. The median total dose area product did not significantly differ between CA and 3DRA in the BA (27.88 μGym^2^/kg versus 15.81 μGym^2^/kg, *p* = 0.275) or stent group (37.34 μGym^2^/kg versus 45.24 μGym^2^/kg, *p* = 0.090). 3DRA was of additional value in 96.8 % of the interventions.

**Conclusions:**

3DRA is superior to CA in diagnostic quality and not associated with increased radiation exposure. It provides high additional value in guiding CoA related interventions.

**Electronic supplementary material:**

The online version of this article (doi: 10.1007/s12471-016-0899-2) contains supplementary material, which is available to authorized users.

## Introduction

Coarctation of the aorta (CoA) is increasingly being treated with percutaneous interventions, as it is equally as safe and effective as surgery [1–5]. Detailed visualisation of the heart, great vessels and surrounding structures is key to a successful intervention [6–8]. However, traditional conventional biplane angiography (CA) only provides a two-dimensional image of the patient’s anatomy [6, 7, 9]. Three-dimensional rotational angiography (3DRA) produces three-dimensional (3D) images and may provide more detailed information [6, 7, 9]. Furthermore, the 3D-image generated can be used as a 3D-roadmap for guiding interventions [6, 7, 9].

Since 3DRA is a relatively new imaging technique in the paediatric cardiac catheterisation laboratory, the available literature on this subject is limited. Previously conducted studies have mainly focused on radiation dose reduction [8, 10, 11] and fail to directly compare diagnostic accuracy and therapeutic success between CA and 3DRA. Furthermore, relatively small and heterogeneous populations were studied [7, 9]. Therefore, the aim of this study is to evaluate the diagnostic and therapeutic additional value of 3DRA compared with CA in percutaneous treatment of children with a CoA. In addition, technical settings of 3DRA are analysed to develop an imaging protocol for optimal visualisation of a CoA at the lowest achievable radiation dose.

## Methods

### Study population

This retrospective study analysed CA and 3DRA-guided cardiac catheterisations that were performed in CoA patients at the Wilhelmina Children’s Hospital, University Medical Center Utrecht between January 2003 and December 2015. All children who underwent cardiac catheterisation for aortic arch interventions were included. For designing the 3D-imaging protocol all children who underwent a 3DRA run during a diagnostic or interventional catheterisation were included. The institutional medical ethics committee provided a waiver for this study.

### Data collection

Demographic, clinical and catheterisation data and technical settings of the 3DRA were retrospectively obtained from patient files, cardiac catheterisation database (Filemaker Pro 11), and image databases (Xcelera 4.1, OsiriX MD 2.6 and Siemens syngoDynaCT).

Fluoroscopy films were included in the analysis, but still frames were excluded. Diagnostic angiographies were defined as all images with contrast displaying relevant cardiac compartments and vessels. Interventional angiographies were defined as all images with contrast displaying balloons or stents and post-interventional control images. In diagnostic angiographies, visibility of the left ventricle, aorta, coronary arteries and ostium of the left subclavian artery was scored on a 3-point scale. Good image quality was defined as providing a clear delineation of cardiovascular structures, moderate image quality as providing an estimated delineation and poor image quality as providing no delineation. Image quality was determined by the primary researcher and in case of doubt by paediatric cardiologists (GK and/or JB).

Therapeutic success was defined as a post-interventional systolic gradient across the CoA under general anaesthesia of <20 mm Hg [12]. Complications were collected in accordance with guidelines [13]. Radiation is reported as dose area product (DAP), which is defined as the product of radiation dose and exposed patient surface [11]. Two paediatric cardiologists (GK and JB) determined the additional value of 3D-images compared with CA images according to a 5-point scale, ranging from essential to misleading [6, 7].

### Data analysis

To analyse radiation, fluoroscopy time and number of angiographies performed, three groups were created: patients with sole diagnostic CAs, both diagnostic CAs and 3DRAs and only diagnostic 3DRAs. Diagnostic quality was analysed in the full population, as this is not influenced by the subsequent type of intervention. Further analyses were performed in patients undergoing either balloon angioplasty (BA) or single stent implantation to enhance comparability. Patients receiving multiple stent implantations were excluded.

3DRA runs of perfect diagnostic quality without artefacts, with the exception of stent artefacts, were used to design the 3D-imaging protocol. Patients with incomplete data sets were excluded.

Data are presented as frequency with percentages of total, mean with standard deviation (SD) or median with interquartile range (IQR). Chi-square and Fisher’s exact tests were used to compare dichotomous variables. Unpaired two-tailed T‑tests and Mann-Whitney U tests were used to compare normally and non-normally distributed continuous variables respectively. A *p*-value <0.05 was considered statistically significant. All analyses were performed using SPSS version 21.

## Results

### Study population

In total, 134 patients underwent 183 catheterisations. Sixty-seven patients underwent BAs, 61 CAs (80.3 %) and 15 3DRAs (19.7 %). Sixty-four patients received stents, 43 CAs (61.4 %) and 27 3DRAs (38.6 %). Median age was 0.52 years in the BA group and 11.19 years in the stent group (*p* < 0.001). In the BA group, maximum Doppler velocity in the descending aorta was significantly lower in the 3DRA group (*p* = 0.020). In the stent group, the 3DRA group were significantly older (*p* = 0.045) (Table 1).

**Table 1:** Baseline characteristics of the patients

Characteristic	Type of catheterisation		
*Balloon angioplasty*	*CA* *n = 61*	*3DRA* *n = 15*	*p-value*
Males	37 (60.7)	8 (53.3)	0.605
Native coarctation, yes	4 (6.6)	3 (20.0)	0.134
Recurrent coarctation, yes	57 (93.4)	12 (80.0)	0.134
Medical history			
– Coarctation	14 (23.0)	3 (20.0)	1.000
– Hypoplastic left heart syndrome or hypoplastic aorta	30 (49.2)	10 (66.7)	0.224
– Interrupted aortic arch	11 (18.0)	2 (13.3)	1.000
– Other	6 (9.8)	0 (0.0)	0.592
Age (years)	0.60 (0.28–1.26)	0.32 (0.25–2.91)	0.493
Height (cm)	68.00 (60.00–80.50)	61.00 (52.00–91.00)	0.518
Weight (kg)	7.40 (5.00–10.00)	5.70 (4.70–12.90)	0.724
Systolic blood pressure right arm (mm Hg)	117.35 ± 15.34	111.92 ± 23.99	0.467
Diastolic blood pressure right arm (mm Hg)	63.98 ± 13.16	58.33 ± 10.63	0.173
Maximum CW Doppler velocity in DAO (m/s)	3.84 ± 0.74	3.30 ± 0.66	0.020
Invasive gradient across the CoA under general anaesthesia (mm Hg)	22.50 (13.25–40.00)	25.00 (10.00–35.00)	0.591
Diameter of CoA (mm)	3.90 (2.70–5.20)	4.90 (3.80–6.80)	0.090
Non-CoA related diagnostic or interventional procedures performed, yes	25 (41.0)	12 (80.0)	0.007
*Stent*	*CA* *n = 43*	*3DRA* *n = 27*	*p-value*
Males	26 (60.5)	17 (63.0)	0.834
Native coarctation, yes	17 (39.5)	10 (37.0)	0.834
Recurrent coarctation, yes	26 (60.5)	17 (63.0)	0.834
Medical history			
– Coarctation	30 (69.8)	18 (66.7)	0.786
– Hypoplastic left heart syndrome or hypoplastic aorta	9 (20.9)	8 (29.6)	0.409
– Interrupted aortic arch	1 (2.3)	1 (3.7)	1.000
– Other	3 (7.0)	0 (0.0)	0.279
Age (years)	9.10 (3.43–13.34)	12.82 (8.78–14.76)	0.045
Length (cm)	141.00 (91.00–157.00)	150.00 (135.00–170.00)	0.101
Weight (kg)	32.80 (14.00–47.80)	44.50 (32.00–55.00)	0.098
Systolic blood pressure right arm (mm Hg)	127.78 ± 16.26	133.59 ± 19.02	0.194
Diastolic blood pressure right arm (mm Hg)	66.94 ± 11.65	73.81 ± 13.07	0.055
Maximum CW Doppler velocity in DAO (m/s)	3.35 ± 0.63	3.24 ± 0.68	0.359
Invasive gradient across the CoA under general anaesthesia (mm Hg)	19.50 (12.25–33.25)	20.00 (13.00–30.00)	0.523
Diameter of CoA (mm)	7.50 (3.88–9.33)	8.10 (4.30–9.80)	0.403
Non-CoA related diagnostic or interventional procedures performed, yes	12 (27.9)	10 (37.0)	0.423

Medical history of coarctation indicates any medical history in which a CoA was the main diagnosis

*CW* continuous wave, *DAO* descending aorta

### Diagnostic value

**Fig. 1:**
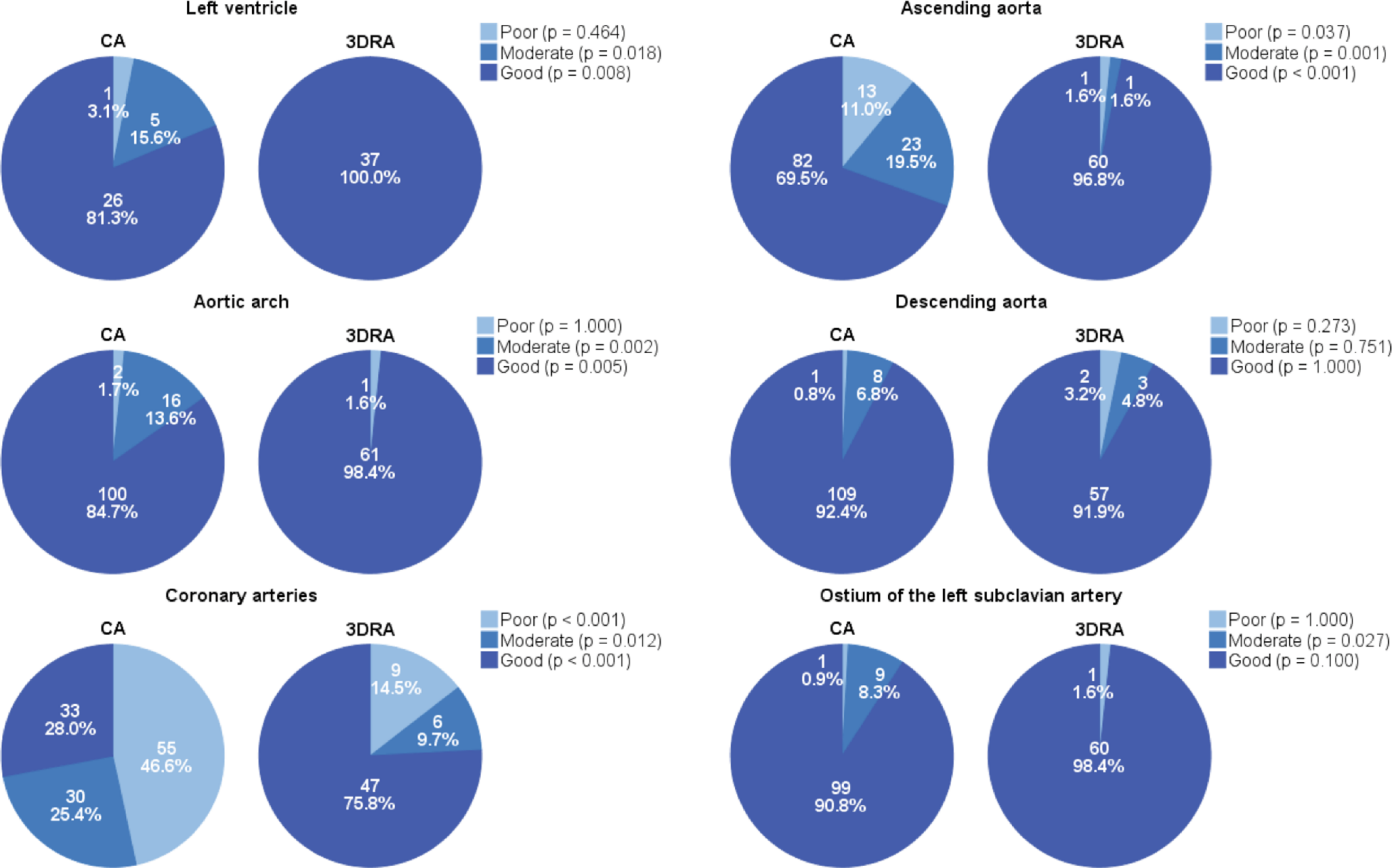
Image quality of relevant cardiovascular structures with CA and 3DRA. Overview of the image quality per cardiac compartment or vessel. The *p*-values display the difference between CA and 3DRA per image quality category

3DRA was superior in displaying the left ventricle, ascending aorta, aortic arch and coronary arteries. 3DRA displayed the descending aorta and ostium of the left subclavian artery equally as well as CA (Fig. 1). Artefacts were significantly more frequently encountered in 3DRAs (25.8 %) than in CAs (5.1 %) (*p* < 0.001). In the 3DRA group, artefacts were mostly caused by catheter movements (*N* = 7 (43.8 %)) due to placement of the angiographic catheter in the ascending aorta (*N* = 5) or aortic arch (*N* = 1).

### Therapeutic value

In the BA group, the 3DRA group had a significantly higher success rate than the CA group: 100.0 % versus 68.9 % respectively (*p* = 0.016). All stent interventions were successful. In the BA group, there was a trend towards lower median residual gradients in the 3DRA than in the CA group (9.00 mm Hg versus 13.00 mm Hg, *p* = 0.087). In the stent group, systolic and diastolic blood pressure were significantly higher in the 3DRA than in the CA group (*p* = 0.002 and *p* = 0.044 respectively) (Table 2).

**Table 2:** Results of the interventions

Characteristic	Type of catheterisation		
*Balloon angioplasty*	*CA* *n = 61*	*3DRA* *n = 15*	*p-value*
Successful, yes	42 (68.9)	15 (100.0)	0.016
Systolic blood pressure right arm (mm Hg)	103.85 ± 16.37	109.08 ± 22.83	0.382
Diastolic blood pressure right arm (mm Hg)	60.77 ± 11.28	56.42 ± 9.49	0.220
Maximum CW Doppler velocity in DAO (m/s)	2.81 ± 0.55	2.56 ± 0.47	0.128
Invasive gradient across the CoA under general anaesthesia (mm Hg)	13.00 (6.75–24.25)	9.00 (3.75–13.00)	0.087
Diameter of CoA (mm)	5.20 (4.15–6.60)	5.70 (4.94–7.78)	0.367
Procedural duration (min)	113.00 (80.00–151.50)	120.00 (105.00–190.00)	0.074
*Stent*	*CA* *n = 43*	*3DRA* *n = 27*	*p-value*
Successful, yes	41 (100.0)	27 (100.0)	NA
Systolic blood pressure right arm (mm Hg)	116.31 ± 14.89	128.17 ± 12.95	0.002
Diastolic blood pressure right arm (mm Hg)	63.10 ± 11.33	69.04 ± 10.35	0.044
Maximum CW Doppler velocity in DAO (m/s)	2.56 ± 0.62	2.52 ± 0.59	0.774
Invasive gradient across the CoA under general anaesthesia (mm Hg)	3.00 (0.00–5.75)	2.50 (0.00–6.50)	0.947
Diameter of CoA (mm)	11.30 (9.00–13.00)	11.20 (9.00–13.80)	0.851
Procedural duration (min)	132.00 (89.00–153.00)	140.00 (105.00–195.00)	0.103

*CW* continuous wave, *DAO* descending aorta, *NA* not applicable

### Complications

In the BA group, procedural complications occurred in 8 CA catheterisations (13.1 %) versus 4 3DRA catheterisations (26.7 %) (*p* = 0.238) and interventional complications in 18 CA catheterisations (29.5 %) versus 3 3DRA catheterisations (20.0 %) (*p* = 0.538). In the stent group, procedural complications occurred in 15 CA catheterisations (34.9 %) versus 7 3DRA catheterisations (25.9 %) (*p* = 0.432) and interventional complications in 1 CA catheterisation (2.3 %) versus 0 3DRA catheterisations (*p* = 1.000). Additional Table 4 displays the different types of complications.

### Angiographies and radiation

3DRA was associated with fewer additional interventional CAs in both the BA and stent group. This difference was significant for the A‑plane angiographies in the stent group (*p* = 0.021). In the BA group, the median total number of additional CAs was 6.00 (4.00–8.00) in the CA, and 2.00 (0.50–2.00) in the 3DRA group (*p* = 0.001). In the stent group, this was 16.00 (12.00–18.00) in the CA, and 8.00 (4.00–9.50) in the 3DRA group (*p* < 0.001) (Additional Table 5).

In the BA group, all DAPs were lower in the 3DRA than in the CA group. In the stent group, DAP due to additional angiographies was significantly lower in the 3DRA than in the CA group (*p* = 0.042) (Table 3).

**Table 3:** Radiation exposure

DAP	Type of catheterisation					
*Balloon angioplasty*	*CA* *n = 61*	*CA and 3DRA* *n = 11*	*p-value* ^a^	*3DRA* *n = 4*	*p-value* ^b^	*p-value* ^c^
Fluoroscopy (μGym^2^/kg)^d^	8.82 (3.35–15.00)	9.07 (7.27–25.39)	0.284	8.54 (3.25–26.44)	0.734	0.514
CA (μGym^2^/kg)^d^	7.34 (1.97–18.55)	5.28 (1.36–9.42)	0.469	1.30 (0.55–10.26)	0.089	0.433
3DRA (μGym^2^/kg)^d^	6.96 (4.64–11.79)^e^	8.61 (6.71–14.06)	0.396	5.97 (3.01–8.16)	0.462	0.151
Total (μGym^2^/kg)	27.88 (16.12–44.11)	22.52 (16.17–45.09)	0.736	15.81 (6.97–44.70)	0.275	0.361
Fluoroscopy time (min)	9.80 (6.55–17.00)	18.50 (13.50–26.20)	0.003	16.75 (10.33–50.18)	0.107	0.794
*Stent*	*CA* *n = 43*	*CA and 3DRA* *n = 14*	*p-value* ^a^	*3DRA* *n = 13*	*p-value* ^b^	*p-value* ^c^
Fluoroscopy (μGym^2^/kg)^d^	12.85 (10.04–21.99)	17.85 (11.15–30.06)	0.614	15.17 (11.84–34.65)	0.243	0.961
CA (μGym^2^/kg)^d^	13.10 (7.95–19.56)	12.38 (4.47–37.82)	0.850	6.56 (2.84–10.18)	0.042	0.145
3DRA (μGym^2^/kg)^d^	NA	22.31 (8.11–34.71)	NA	22.17 (15.23–30.54)	NA	0.923
Total (μGym^2^/kg)	37.34 (25.93–59.77)	48.90 (36.04–107.25)	0.072	45.24 (37.78–81.34)	0.090	0.923
Fluoroscopy time (min)	16.10 (11.38–20.28)	23.90 (15.20–35.80)	0.011	20.40 (13.90–36.80)	0.047	0.771

^a^ *P*-value that indicates the difference between CA and CA and 3DRA.

^b^ *P*-value that indicates the difference between CA and 3DRA.

^c^ *P*-value that indicates the difference between CA and 3DRA and 3DRA.

^d^ These subgroups only became available from September 2011.

^e^ The DAP for 3DRA in the CA group was due to post-interventional 3DRA runs.

*DAP* dose area product, *NA *not applicable.

### Additional value of 3DRA

**Fig. 2:**
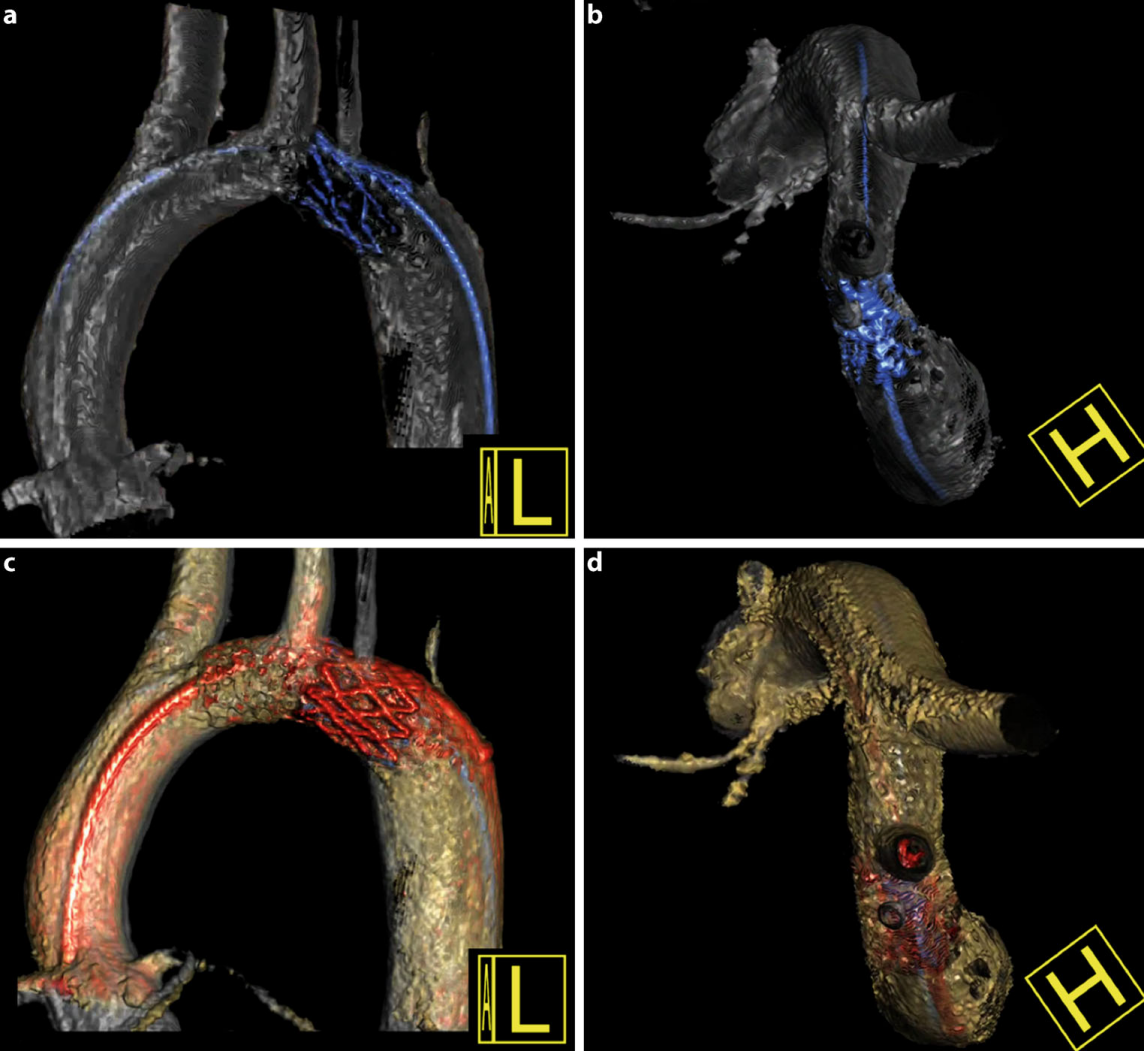
Understanding coarctation morphology with 3DRA. A 17.5-year-old patient with aortic arch hypoplasia made visible with a cranial view from the 3DRA (**b**), but not with the lateral view (**a**), which led to stenting of the transverse aortic arch (**c**,**d**)

**Fig. 3:**
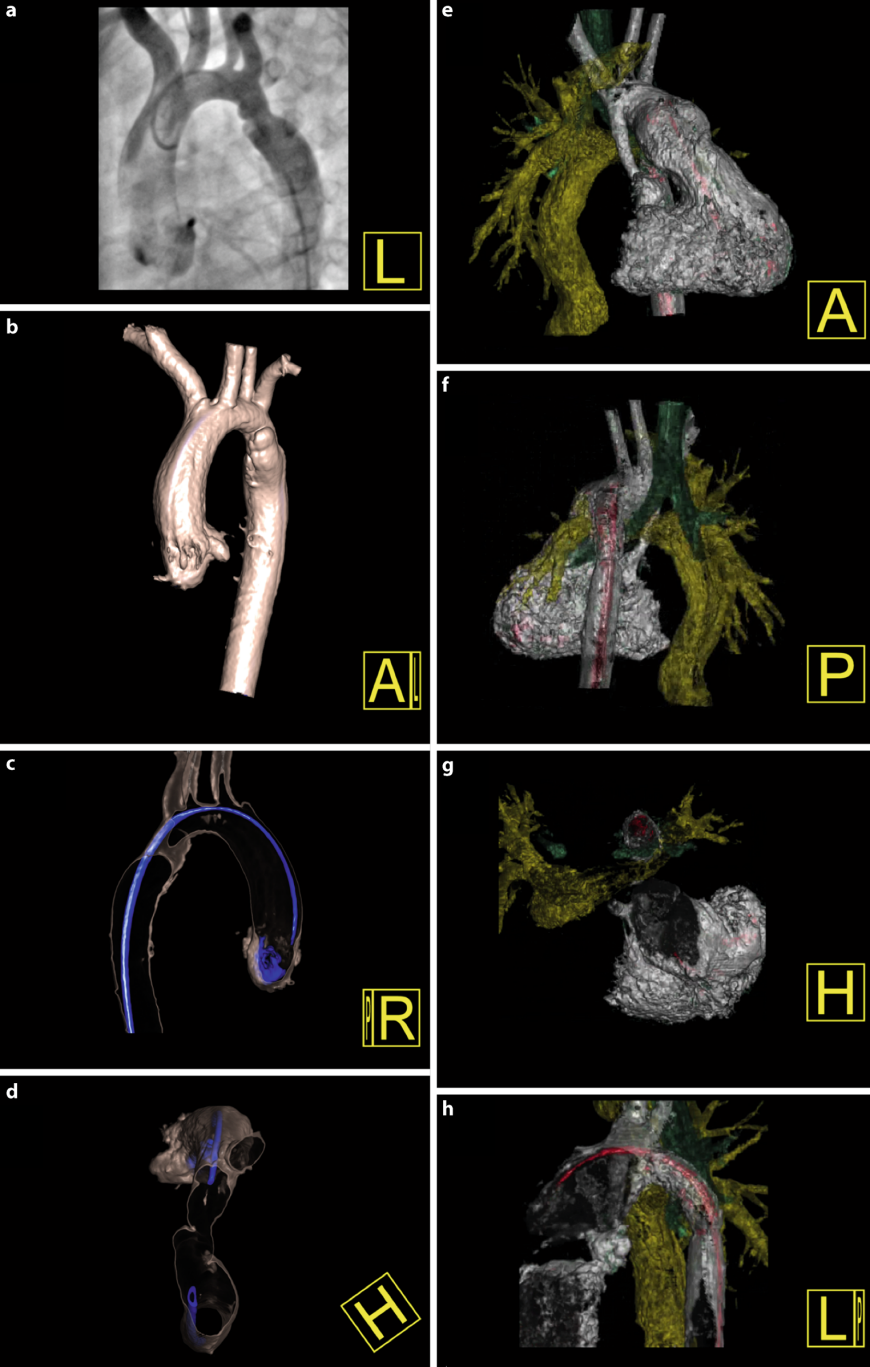
Displaying other vascular and extravascular structures with 3DRA. The left side displays a 2.5-year-old patient with a recurrent coarctation. 3DRA displayed a dissection on the anterior (**b**), lateral (**c**) and superior view (**d**), which was not clearly visible on the CA (**a**), leading to the decision of a stent implantation. The right side displays a 3.5-year-old patient with a recoarctation and a univentricular heart. 3DRA displayed an important interaction between the coarctation stent (white), the left pulmonary artery stent (yellow) and the left bronchus (green), which can be visualised from multiple angles (**e**-**h**)

The additional value of 62 3DRAs in CoA interventions was evaluated. The scores were as follows: 10 3DRAs were essential (16.1 %), 44 very useful (71.0 %), 6 useful (9.7 %), 2 not useful (3.2 %) and 0 misleading (0.0 %). Not useful 3DRAs were of poor diagnostic quality due to suboptimal settings of the 3DRA. The greatest benefit of 3DRA was the ability to fully understand aortic arch and CoA morphology (*N* = 60 (96.8 %)) (Fig. 2). Other benefits were the possibility of displaying other cardiovascular structures (*N* = 17 (27.4 %)) and vascular anomalies (*N* = 17 (27.4 %)) in the same run (Fig. 3). In univentricular heart patients, 3DRA was useful in displaying vessel-vessel or vessel-bronchi interactions (*N* = 5 (8.1 %)) (Fig. 3).

Live fluoroscopy overlay of the 3D-reconstructed image (iPilot) was used in 43 catheterisations (69.4 %). The iPilot was very helpful in correctly positioning guidewires and devices. No anatomy shifts occurred due to insertion of stiff wires. However, small discrepancies between the vessel and device did occur (*N* = 6 (14.0 %)).

### Learning curve

The total number of 3DRA-guided CoA catheterisations increased from 12 in 2012 to 23 in 2015. Conversely, the median total DAP strongly decreased from 87.06 μGym^2^/kg to 23.20 μGym^2^/kg in the BA group and from 90.15 μGym^2^/kg to 43.01 μGym^2^/kg in the stent group.

### 3D imaging protocol

**Fig. 4:**
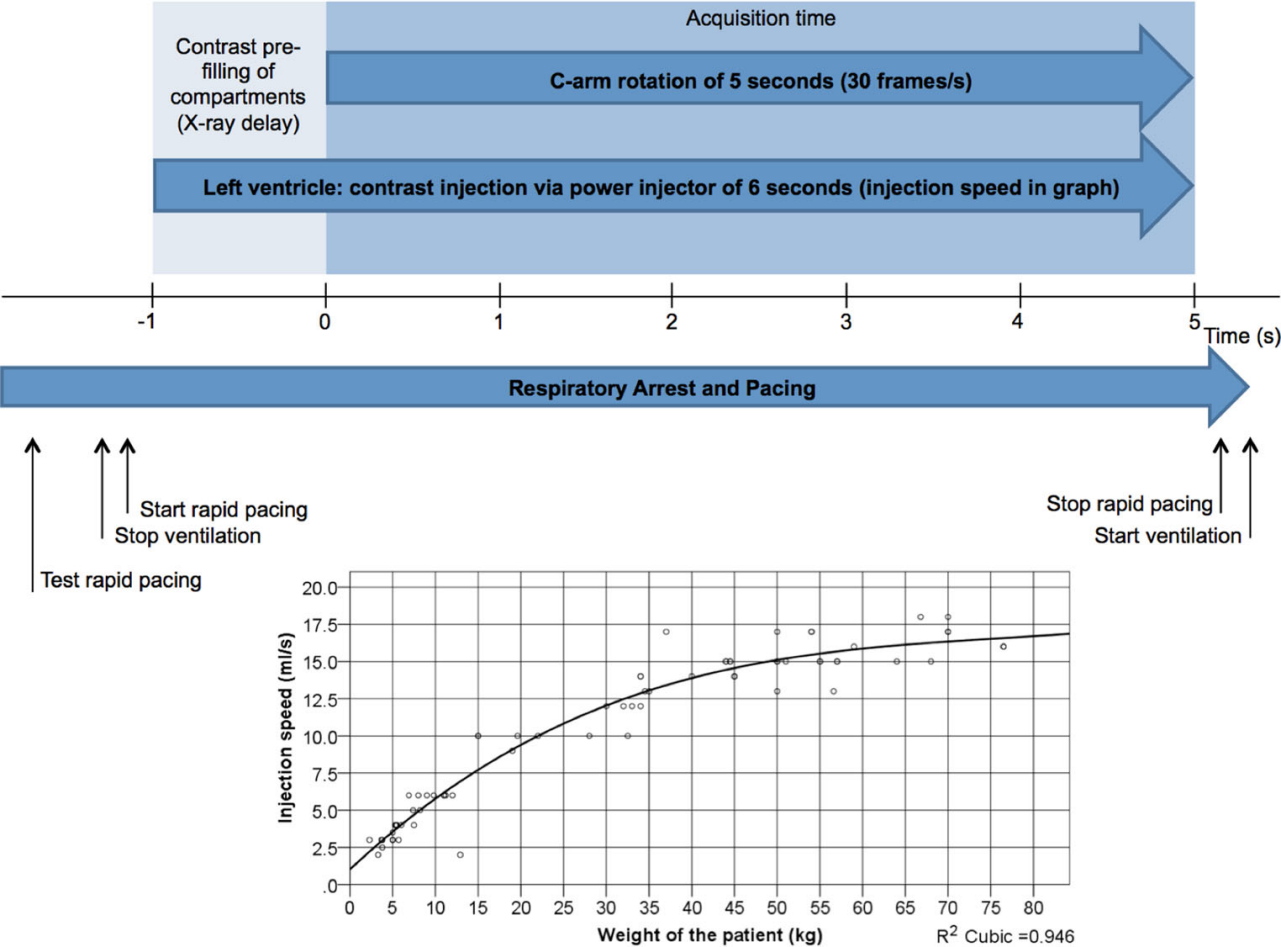
Workflow of the 3DRA run. 3DRAs were performed with Artis Zee biplane (Siemens, Erlangen, Germany) under breath hold and rapid pacing in the right ventricle (*N* = 58 (90.6 %)). Pacing frequency was increased from 160 beats/min upwards to achieve a 50 % reduction in systolic blood pressure to allow optimal contrast filling. Maximal zoom and collimation were applied. The contrast was diluted to a 2:1 contrast:saline ratio and administered with a power injector prior to the compartment of interest; mostly the left ventricle (*N* = 58 (81.7 %)). Images were recorded with a frame rate of 60 frames/s or 30 frames/s (*N* = 61 (85.9 %))

77 patients received 126 3DRA runs. Two 3DRA runs were performed for pre- and post-procedural imaging (*N* = 28), technical difficulties (*N* = 4) and requirement of re-intervention (*N* = 1). Seventy-one 3DRAs were suitable for designing the imaging protocol (Fig. 4).

## Discussion

3DRA is increasingly being used in the catheterisation laboratory [6–11, 14]. However, the available literature is limited in quantity and diversity. This study aims to evaluate the diagnostic and therapeutic additional value of 3DRA compared with CA in the percutaneous treatment of CoAs. Additionally, an imaging protocol was designed to achieve 3D-images of optimal diagnostic quality with the lowest radiation dose possible. Overall, 3DRA proved superior to CA in terms of diagnostic value and comparable to CA in terms of success, complications and radiation exposure. The additional value of 3DRA arises primarily from full understanding of aortic arch and CoA morphology.

### Diagnostic value

Image quality of the left ventricle, ascending aorta, aortic arch and coronary arteries is significantly higher in 3DRA than in CA. These structures are important for good device positioning. Studies with varying areas of interest report diagnostic quality rates of 3DRA from 71 to 94 % [8, 9, 14]. The diagnostic quality in this study is even higher at 98.4 % good image quality of the aortic arch and subclavian artery. This could be due to consistent use of rapid pacing and X‑ray delay to ensure optimal contrast filling [8, 9, 14]. Image quality was similar in the BA and stent groups despite the significant age difference. However, 3DRA reconstructions contain more artefacts than CA. Some artefacts can be resolved by changing the technical settings of 3DRA and post-processing. In all other, the dynamic rotational angiogram is almost always diagnostic.

### Therapeutic value

Therapeutic success was high in both the BA and stent groups. 3DRA-guided BA resulted in lower residual gradients than previously reported (9.00 versus 12.5 mm Hg) [4, 15] and was superior to CA in our study. This may be the result of superior diagnostic imaging leading to improved interventional therapy or more aggressive treatment strategies. Residual gradients after stenting were low and comparable to other studies in both CA and 3DRA group [4, 16]. To date, no study we are aware of has performed similar comparisons.

Interventional complication rates are comparable to those described elsewhere [4, 13]. Only one stent dislocation occurred in the CA group. One can speculate that additional 3D-imaging helps to optimise stent sizing and positioning, thereby reducing the risk of stent dislocation.

### Angiographies and radiation

In five years, a threefold decrease in radiation exposure was accomplished during 3DRA procedures. There were several reasons for this: first, technical settings were altered and frame rate was lowered from 60 to 30 frames/s without disadvantageous effect on image quality. Second, 3D-images are of superior diagnostic quality which reduces the need for additional CAs. Third, during the learning curve, paediatric cardiologists initially performed both CA and 3DRA, but with increasing knowledge and confidence, this was abandoned.

The radiation dose in the 3DRA group was not significantly higher than that in the CA group (*p* = 0.275 for BAs; *p* = 0.090 for stents). This contrasts with the study from Manica et al., which reported a significantly higher DAP of a single 3DRA run compared with three CAs in patients under 45 kg [8]. Total DAPs were comparable to other studies, which reported median DAPs for various congenital heart disease interventions of 3605 μGym^2^ [11] and 1429.6 μGym^2^ for CoA stenting [7]. More importantly, we see a clear decrease in DAP, currently resulting in very low total DAPs compared with benchmarks (median) of 2000 μGym^2^ in children between 1–4 years old and 9600 μGym^2^ in children between 10–15 years old [17]. The remaining radiation difference between BA and stenting is due to a difference in complexity of the procedure and age and therefore size of the patients.

### Additional value

3DRA was of additional value in 96.8 % of the interventions, which is similar to other cohorts. However, we found more essential 3DRAs [6, 7]. This can be caused by obtaining more information from 3DRA, as experience with this technique increases.

The greatest advantage of 3DRA is the ability to fully understand aortic arch and CoA morphology. 3DRA is able to visualise the aorta from an infinite number of angles, some of which are very important, such as the cranioposterior angle, which are not provided by CA [6–9]. Furthermore, other vascular and extravascular (e. g. airway) structures can be visualised simultaneously with a high spatial resolution [8]. Moreover, iPilots are of great additional value for guidewire and stent positioning, thereby reducing the need to perform multiple CAs to guide the procedures [6, 7, 9].

### Imaging protocol

We used 3DRA runs of perfect diagnostic quality to design a 3DRA imaging protocol for CoAs. This is the first practical guide ever on optimisation of 3DRA and may help others to start up 3DRA catheterisation laboratories with faster learning curves.

### Limitations

This study is limited by its retrospective nature. In addition, our learning curve influenced some median values, such as radiation doses. Finally, scoring of image quality and additional value of 3DRA cannot be purely objective. However, we tried to limit the subjectivity by using predefined scores.

## Conclusion

3DRA provides rotatable, high spatial resolution 3D-images of superior diagnostic quality to CA. Furthermore, interventions can be effectively and safely performed without exposing patients to higher radiation doses. Finally, iPilots are of additional value in guiding interventions. Therefore, 3DRA should be considered as the standard imaging technique in percutaneous interventions in CoAs.

## Caption Electronic Supplementary Material

Additional Tab. 4 Number and type of complications

Additional Tab. 5 Number of additional conventional angiographies used for CoA related interventions
