# Supplementary material for: Three-dimensional rotational angiography in children with an aortic coarctation
Source: Neth Heart J. 2016 Sep 22;24(11):666–74. doi: 10.1007/s12471-016-0899-2 (PMC5065539; doi:10.1007/s12471-016-0899-2)
Supplement: Supplementary file 1 — Additional Tab. 4 Number and type of complications [file 12471_2016_899_MOESM1_ESM.doc]

**Additional Tab. 1** Number and type of complications

|  | **Balloon angioplasty** | | **Stent** | |
| --- | --- | --- | --- | --- |
| **Type of complication** | **Type of catheterisation** | | **Type of catheterisation** | |
|  | **CA**  ***n* = 8** | **3DRA**  ***n* = 4** | **CA**  ***n* = 16** | **3DRA**  ***n* = 9** |
| *Procedural complications* | N (%) | N (%) | N (%) | N (%) |
| Haematoma | 3 (37.5) | 0 (0.0) | 6 (37.5) | 4 (44.4) |
| Re-bleed | 0 (0.0) | 1 (25.0) | 6 (37.5) | 1 (11.1) |
| Occluded femoral artery | 4 (50.0) | 0 (0.0) | 1 (6.3) | 0 (0.0) |
| Rhythm changes | 1 (12.5) | 0 (0.0) | 0 (0.0) | 2 (22.2) |
| Heart block resolved | 0 (0.0) | 1 (25.0) | 0 (0.0) | 0 (0.0) |
| ST-wave changes | 0 (0.0) | 0 (0.0) | 2 (12.5) | 1 (11.1) |
| Decreased haemoglobin | 0 (0.0) | 1 (25.0) | 0 (0.0) | 0 (0.0) |
| Hypoxia | 0 (0.0) | 1 (25.0) | 0 (0.0) | 0 (0.0) |
| Unplanned extubation | 0 (0.0) | 0 (0.0) | 1 (6.3) | 0 (0.0) |
| Resuscitation | 0 (0.0) | 0 (0.0) | 0 (0.0) | 1 (11.1) |
|  |  |  |  |  |
|  | **CA**  ***n* = 18** | **3DRA**  ***n* = 3** | **CA**  ***n* = 1** | **3DRA**  ***n* = 0** |
| *Interventional complications* | N (%) | N (%) | N (%) | N (%) |
| Intravascular tear | 9 (50.0) | 0 (0.0) | 0 (0.0) | 0 (0.0) |
| Aneurysm | 6 (33.3) | 0 (0.0) | 0 (0.0) | 0 (0.0) |
| Dissection | 1 (5.6) | 2 (66.7) | 0 (0.0) | 0 (0.0) |
| Balloon rupture | 1 (5.6) | 1 (33.3) | 0 (0.0) | 0 (0.0) |
| Need for re-intervention | 1 (5.6) | 0 (0.0) | 0 (0.0) | 0 (0.0) |
| Stent dislocation | NA | NA | 1 (100.0) | 0 (0.0) |

*NA* not applicable.
