# Supplementary material for: Three-dimensional rotational angiography in children with an aortic coarctation
Source: Neth Heart J. 2016 Sep 22;24(11):666–74. doi: 10.1007/s12471-016-0899-2 (PMC5065539; doi:10.1007/s12471-016-0899-2)
Supplement: Supplementary file 2 — Additional Tab. 5 Number of additional conventional angiographies used for CoA related interventions [file 12471_2016_899_MOESM2_ESM.doc]

**Additional Tab. 2** Number of additional conventional angiographies used for CoA related interventions

| **Number of angiographies** | **Type of catheterisation** | | | | | |
| --- | --- | --- | --- | --- | --- | --- |
| Balloon angioplasty | CA  ***n*** = 61 | CA and 3DRA  ***n*** = 11 | p-value^a^ | 3DRA  ***n*** = 4 | p-value^b^ | p-value^c^ |
| Diagnostic A-plane (n) | 2.00 (1.00-2.00) | 2.00 (1.00-2.00) | 0.889 | 0.00 (0.00-0.00) | < 0.001 | 0.007 |
| Diagnostic B-plane (n) | 2.00 (1.00-2.00) | 1.00 (1.00-2.00) | 0.720 | 0.00 (0.00-0.00) | < 0.001 | 0.006 |
| Interventional A-plane (n) | 1.00 (1.00-2.00) | 1.00 (1.00-2.00) | 0.529 | 1.00 (0.25-1.00) | 0.079 | 0.212 |
| Interventional B-plane (n) | 1.00 (1.00-2.00) | 1.00 (1.00-2.00) | 0.294 | 1.00 (0.25-1.00) | 0.059 | 0.374 |
| Total (n) | 6.00 (4.00-8.00) | 6.00 (4.00-10.00) | 0.672 | 2.00 (0.50-2.00) | 0.001 | 0.012 |
|  |  |  |  |  |  |  |
| **Stent** | **CA**  ***n* = 43** | **CA and 3DRA**  ***n* = 14** | **p-value^a^** | **3DRA**  ***n* = 13** | **p-value^b^** | **p-value^c^** |
| Diagnostic A-plane (n) | 3.00 (2.00-4.00) | 2.00 (1.00-3.00) | 0.073 | 0.00 (0.00-0.00) | < 0.001 | < 0.001 |
| Diagnostic B-plane (n) | 3.00 (2.00-4.00) | 1.50 (1.00-3.00) | 0.085 | 0.00 (0.00-0.00) | < 0.001 | < 0.001 |
| Interventional A-plane (n) | 4.00 (3.00-6.00) | 3.50 (2.00-4.25) | 0.077 | 2.00 (1.00-5.00) | 0.021 | 0.432 |
| Interventional B-plane (n) | 5.00 (3.00-6.00) | 3.50 (2.00-5.25) | 0.082 | 4.00 (2.50-5.50) | 0.169 | 0.768 |
| Total (n) | 16.00 (12.00-18.00) | 10.00 (7.00-14.50) | 0.007 | 8.00 (4.00-9.50) | < 0.001 | 0.036 |

^a^ P-value that indicates the difference between CA and CA and 3DRA.

^b^ P-value that indicates the difference between CA and 3DRA.

^c^ P-value that indicates the difference between CA and 3DRA and 3DRA.
